# Tocotrienol-Rich Fraction from Rice Bran Demonstrates Potent Radiation Protection Activity

**DOI:** 10.1155/2015/148791

**Published:** 2015-08-26

**Authors:** Kimberly J. Krager, E. Nathalie Pineda, Sujay V. Kharade, Mary Kordsmeier, Luke Howard, Philip J. Breen, Cesar M. Compadre, Martin Hauer-Jensen, Nukhet Aykin-Burns

**Affiliations:** ^1^Division of Radiation Health, . University of Arkansas for Medical Sciences, 4301 West Markham, No. 522-10, Little Rock, AR 72205, USA; ^2^Department of Pharmaceutical Sciences, University of Arkansas for Medical Sciences, 4301 West Markham, No. 522-10, Little Rock, AR 72205, USA; ^3^Department of Food Science, University of Arkansas, Fayetteville, AR 72704, USA; ^4^Surgical Service, Central Arkansas Veterans Healthcare System, Little Rock, AR 72205, USA

## Abstract

The vitamin E analogs *δ*-tocotrienol (DT3) and *γ*-tocotrienol (GT3) have significant protective and mitigative capacity against the detrimental effects of ionizing radiation (IR). However, the expense of purification limits their potential use. This study examined the tocotrienol-rich fraction of rice bran (TRFRB) isolated from rice bran deodorizer distillate, a rice oil refinement waste product, to determine its protective effects against IR induced oxidative damage and H_2_O_2_. Several cell lines were treated with tocotrienols or TRFRB prior to or following exposure to H_2_O_2_ or IR. To determine the radioprotective capacity cells were analyzed for morphology, mitochondrial bioenergetics, clonogenic survival, glutathione oxidation, cell cycle, and migration rate. TRFRB displayed similar antioxidant activity compared to pure tocotrienols. Cells pretreated with TRFRB or DT3 exhibited preserved cell morphology and mitochondrial respiration when exposed to H_2_O_2_. Oxidized glutathione was decreased in TRFRB treated cells exposed to IR. TRFRB reversed mitochondrial uncoupling and protected cells migration rates following IR exposure. The protective antioxidant capacity of TRFRB treated cells against oxidative injury was similar to that of purified DT3. TRFRB effectively protects normal cells against IR induced injury suggesting that rice bran distillate may be an inexpensive and abundant alternate source.

## 1. Introduction

Radiation is commonly used in the treatment of a wide range of malignancies. More than 50% of cancer patients receive radiotherapy alone or in combination with chemotherapy or surgery in order to achieve local or regional control of their malignancies. Although ionizing radiation (IR) is very effective in killing cancer cells, patients continue to suffer from acute and chronic adverse side effects that limit the therapeutic window for radiotherapy [1]. In addition, significant growth in nuclear power production and radionuclide usage increases the risk of radiation exposure to large populations. Thus, there is a growing need for a safe and effective radioprotector/radiomitigator compound that will minimize the side effects of radiotherapy on normal tissues and reduce the morbidity and mortality from radiation exposure due to accidents or terrorism.

IR exposure causes significant increases in generation of reactive oxygen and nitrogen species (ROS/RNS), such as superoxide, hydrogen peroxide (H_2_O_2_), and peroxynitrite. Alterations in redox homeostasis caused by these reactive species damage the DNA and intracellular compartments leading to perturbations in biochemical reactions and critical pathways in both the short and long term following IR exposure [2–7]. However studies using various antioxidants to counteract the adverse effects of IR in normal tissues were not fruitful, providing only partial protection [8, 9]. Tocotrienols, especially the *γ*- and *δ*-vitamers, are among the few most promising compounds [10–15] and have shown striking effects in protecting against radiation damage. A single injection of *δ*-tocotrienol (DT3) has a dose reduction factor (DRF) of 1.27 as a radioprotectant and 1.1 as a radiomitigator, while *γ*-tocotrienol (GT3) has a DRF of 1.29 as a radioprotector [15]. In addition, these tocols are nontoxic at effective doses [16]. Studies suggest numerous mechanisms by which GT3 and DT3 exert their radioprotective effects, including the enhancement of eNOS activity via regulation of tetrahydrobiopterin availability and prevention of DNA damage to hematopoietic stem and progenitor cells via stimulation of mTOR survival pathways [17–20]. Unfortunately both compounds are in short supply and very expensive.

Compared to the expensive procedure of synthesizing pure tocotrienols, the natural products, for example, palm oil, containing tocol-rich fractions, have been used to supply the radioprotective constituents. Rice bran oil deodorizer distillate (RBODD) is a by-product from the process of refining rice and is usually discarded. It is rich in tocols, including the active *γ*- and *δ*-tocotrienols. It also contains squalene, which has been shown to possess radioprotective capacity [21].

The objective of this research was to characterize the tocotrienol-rich fraction of rice bran (TRFRB) isolated from rice bran oil deodorizer distillate (RBODD) and investigate its protective effects against H_2_O_2_ and IR induced oxidative damage. We used various murine and human cells to assess the efficacy of TRFRB against IR injury by measuring cell morphology, lipid peroxidation, thiol oxidation, cellular bioenergetics and mitochondrial respiration, keratinocyte migration, cell cycle analysis, and clonogenic cell survival.

## 2. Materials and Methods

### 2.1. Cell Culture

H9c2 rat cardiomyocytes and immortalized human skin keratinocyte cell line (HaCaT) were kindly donated by Dr. Marsh (UAMS) and Dr. Domann (UIOWA), respectively. Primary human dermal fibroblasts were purchased from Lifeline Cell Technologies (Frederick, MD) and rat liver microsomes were purchased from BD Biosciences. All cell lines were grown in Dulbecco's Minimal Essential Medium containing high glucose and supplemented with 1 mM sodium pyruvate (Gibco), 10% FBS (HyClone), and 1%* L*-glutamine (Gibco) in the presence of 1% penicillin and streptomycin. Cells were maintained and experiments were accomplished in a humidified incubator at 37°C with 5% CO_2_. For mitochondrial respiration studies, culture media in cells were changed to unbuffered DMEM supplemented with 4 mM glutamate and incubated in a non-CO_2_ incubator for 1 h at 37°C, before they were placed in XF96 Extracellular Flux Analyzer. In all experiments, where DMSO was used as vehicle, its final concentration in the tissue culture dishes was kept at 0.1% or less (v/v).

### 2.2. Preparation of TRFRB (Tocotrienol-Rich Fraction of Rice Bran)

TRFRB was prepared by rice bran oil deodorizer distillate (RBODD) by an adaptation of the method described by Ko et al. [22]. RBODD, provided by Riceland Foods (Stuttgart, AR), was refluxed with acetonitrile in a 1 : 10 ratio. After refluxing, samples were cooled to ambient temperature and stored at −20°C for 24 h to precipitate the cold insoluble sterols from the soluble tocols. The sterols were filtered using a sintered glass filter, the filtrate was collected, and the solvent evaporated using a SpeedVac concentrator. Samples were then mixed with an ethanolic solution containing 5% (w/v) pyrogallol and the mixture was refluxed to boiling. Tocols were saponified at 70°C for 30 min following the addition of 1 mL of 50% (w/v) aqueous potassium hydroxide solution. After cooling in an ice water bath the mixture was transferred to a 500 mL separatory funnel, and 30 mL diethyl ether and 20 mL distilled water were added. The diethyl ether extraction was repeated two times and the ether fractions were pooled. The pooled diethyl ether was washed three times with 20 mL distilled water and then filtered through anhydrous sodium sulfate for 30 min to remove any excess water. The diethyl ether was evaporated using the SpeedVac concentrator to obtain the tocotrienol-rich fraction of rice bran (TRFRB).

### 2.3. Qualitative and Quantitative Analysis of TRFRB

Composition of TRFRB was determined by a gas chromatography/mass spectrometry (GC/MS) method. For this analysis, samples of the extract were analytically transferred to deactivated glass microinserts using methylene chloride, dried under nitrogen, and derivatized using* N*-methyl-*N*-TMS-trifluoroacetamide (Restek Co., Bellefonte, PA) at 25°C. Analyses were performed by GC/MS using Agilent 5975 GC/MSD (Agilent Technologies, Santa Clara, CA). The GC was equipped with a 30 m HP-5MS column (0.250 mM, 0.25 *μ*M). Samples were analyzed using helium as the carrier gas (head pressure of 27 psi) and 1 *μ*L splitless injection; the injector temperature was 275°C; the column temperature was maintained at 220°C for 2 min followed by a gradient of 25°C/min to 300°C, remaining at that temperature for 10 min. The transfer line temperature was maintained at 285°C for 13.5 min followed by a gradient of 25°C/min to 300°C, remaining at that temperature for 10 min. The MS conditions were as follows: electron impact, source temperature 230°C, quadrupole temperature 150°C, and ionization voltage 70 eV. The identity of the tocols in the rice extract was established by comparing the retention time and mass spectra of authentic samples of each tocol (Yasoo Health, Jonesborough, TN). Quantitation of the derivatized tocols was performed in triplicate using single-ion monitoring. The MS ions detected were 496.8 (*α*-tocotrienol), 502.9 (*α*-tocopherol), and 482.8 (*β*-*γ*-tocotrienol). The confirming ions were 237, 237, and 223, respectively. All the bioassays were conducted using proper dilutions of stock solution of 76.2 mg/mL TRFRB in DMSO standardized to 20 mM content of tocotrienols (9.8 mM of GT3/BT3 and 8.6 mM of AT3).

### 2.4. TBARS Assay

The antioxidant activity of TRFRB was assessed measuring the ability to prevent microsomal lipid peroxidation. For this a TBARS assay was performed using a modified version of the original method described by Buege and Aust (1978). Briefly, rat liver microsomes (BD Biosciences) were suspended in PBS in glass tubes at a final concentration of 1 mg/mL. To this, increasing concentrations of TRFRB dissolved in DMSO were added. DMSO alone was used as control. The final DMSO percentage was kept under 0.1%. Microsomes were subsequently incubated with the test compounds at 37°C for one hour in a shaking water bath (100 rpm). After one hour, freshly made* tert*-butyl hydroperoxide (TBHP) solution was added to microsomes at a final concentration of 200 *μ*M and incubation was continued for another 30 min. The reaction was terminated by addition of 20% trichloroacetic acid and tubes were placed on ice for 15 min. Equal volume of 0.67% thiobarbituric acid in 0.05 N NaOH was added and the final mixture was heated at 95°C for 45 min to allow the color to develop. Finally, the tubes were cooled on ice and centrifuged at 1000 rpm for 20 min and the supernatant was read at 532 nm. The percent TBARS was calculated using a standard curve obtained using malondialdehyde (MDA). The results in Figure 2 are expressed in *μ*M of tocotrienols and compared with the effects of pure gamma-tocotrienol [23].

### 2.5. Cell Morphology

H9c2 rat heart cardiomyocytes were treated with either vehicle control (DMSO, 0.1% v/v), 5 *μ*M delta-tocotrienol, or 5 *μ*M TRFRB overnight. Cells were then exposed to 100 or 200 *μ*M H_2_O_2_ for 4 h. Morphological changes were recorded under an inverted microscope. Pictures were taken at 10x magnification. Scale bar represents 400 *μ*m.

### 2.6. Mitochondrial Bioenergetics

Oxygen consumption rate (OCR) was measured at 37°C using an XF96 Extracellular Analyzer (Seahorse Bioscience) as previously described [24]. H9c2 rat heart cardiomyocytes cells were plated in 96-well Seahorse plates. The next day they were treated with either vehicle (DMSO, 0.1% v/v) or 5 *μ*M of DT3 or 5 *μ*M of TRFRB overnight. The cells were then treated with 50 or 100 *μ*M H_2_O_2_ for 4 h at 37°C. The media in the wells were changed to unbuffered DMEM supplemented with 4 mM glutamate and incubated in a non-CO_2_ incubator for 1 h at 37°C. Three baseline measurements were acquired before injection of mitochondrial inhibitors or uncouplers. Readings were taken after sequential addition of oligomycin (10 *μ*M), carbonylcyanide 4-(trifluoromethoxy)phenylhydrazone (FCCP, 10 *μ*M), and rotenone/antimycin A (10 *μ*M). Oxygen consumption rates were calculated by the Seahorse XF96 software and represent an average of 3 measurements on 8 different wells. The rate of measured oxygen consumption was reported as pmole O_2_ consumed per minute per 10,000 cells.

In order to determine the radioprotective effects of TRFRB, human immortalized keratinocytes (HaCaT cells) were plated in 96-well Seahorse plates. The next day they were treated with either vehicle (DMSO, 0.1% v/v) or 5 *μ*M of DT3 or 5 *μ*M of TRFRB overnight. The Seahorse plates were either sham irradiated or irradiated with 8 Gy of *γ*-irradiation. Both plates were sequentially run in the XF96 Extracellular Analyzer as described above.

### 2.7. *In Vitro* Scratch Assay

Human immortalized keratinocytes (HaCaT cells) were plated in 12-well culture plates and irradiated with 8 Gy or sham irradiated 24 h after plating. The cells were given either vehicle (DMSO, 0.1% v/v) or 5 *μ*M of DT3 or 5 *μ*M of TRFRB 4 h following radiation. Culture media were replaced again at 36 h following radiation. At the 72 h time point, a scratch was made using a sterile micropipette tip in each well. This created an open area into which the keratinocytes migrated. Two areas of the scratch margins were imaged daily until scratches were closed. The area of the scratch remaining was measured on the images at five points per field, with two fixed fields averaged using Cell Analyzer 1.0 (BWTech, Iowa City, IA). Measurements were stopped when cells no longer made positive progress towards the scratch origin [25].

### 2.8. Clonogenic Cell Survival

Human immortalized keratinocytes (HaCaT cells) were plated and irradiated with 8 Gy or sham irradiated 24 h after plating. The cells were given either vehicle (DMSO, 0.1% v/v) or 5 *μ*M of DT3 or 5 *μ*M of TRFRB 4 h following radiation. Culture media were replaced again at 36 h following radiation. At the 72 h time point, the cells were trypsinized and plated at various dilutions for clonogenic survival. Cells were fixed in 70% ethanol and stained with Coomassie Blue 14 d later. Clones with more than 50 cells were counted under dissecting microscope [26].

### 2.9. Measurements of Glutathione and Glutathione Disulfide Levels

Human dermal fibroblasts were plated in 100 mm plates and the next day they were treated with vehicle (DMSO, 0.1% v/v) or 5 *μ*M of TRFRB for 16 h. The cells were then irradiated with 8 Gy. Cell pellets were collected 24 h following irradiation and pipette-homogenized in 50 mM PO_4_ buffer pH 7.8 containing 1.34 mM diethylenetriaminepentaacetic acid (DETAPAC buffer). Total glutathione content was determined as previously described using the Anderson method [26, 27]. To distinguish reduced glutathione (GSH) and glutathione disulfide (GSSG), 2 *μ*L of a 1 : 1 mixture of 2-vinylpyridine and ethanol was added per 30 *μ*L of sample and assayed as described previously [26, 28]. All glutathione determinations were normalized to the protein content of whole homogenates using the Lowry et al. method [29].

### 2.10. Cell Cycle Analysis

Human dermal fibroblasts were plated in 100 mm plates and the next day they were treated with vehicle (DMSO, 0.1% v/v) or 5 *μ*M of TRFRB overnight. The cells were then irradiated with 2, 4, 6, or 8 Gy. Cell pellets were collected 24 h following irradiation and fixed in 70% ethanol. Then the ethanol-fixed cells pellets were washed with PBS and treated with RNase A for 30 min followed by staining with propidium iodide (35 *μ*g/mL). DNA content of propidium iodide-stained cells was analyzed by flow cytometry, and the percentage of cells in each phase of the cell cycle was calculated [2].

### 2.11. Statistical Analysis

Statistical analysis was performed using GraphPad Prism 5.0 (GraphPad Software, San Diego, California). Data were expressed as mean ± SD unless otherwise specified. One-way ANOVA analysis with Tukey's post analysis was used to study the differences among 3 or more means. Significance was determined at *p* < 0.05 and the 95% confidence interval.

## 3. Results

**Figure 1:**
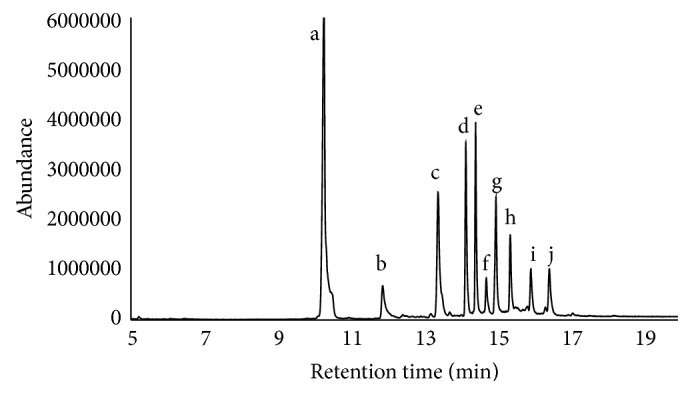
Composition of TRFRB determined by GC/MS. (a) Squalene, (b) *δ*-tocopherol, (c) *β*- and *γ*-tocopherol, (d) *α*-tocopherol, (e) *β*- and *γ*-tocotrienol, (f) campesterol, (g) *α*-tocotrienol, (h) *β*-sitosterol, (i) cycloartenol, and (j) 2,4-methylenecycloartenol.

A full scan GC/MS analysis demonstrated approximately 17.3% of tocotrienols, 25.3% of tocopherols, and 31.2% of squalene (Figure 1, Table 1).

**Figure 2:**
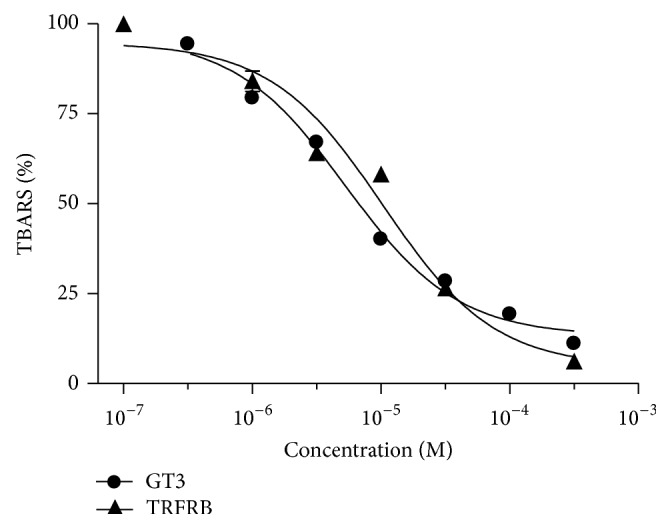
Comparative antioxidant activity of pure *γ*-tocotrienol (GT3) and TRFRB was determined by TBARS analysis.

In order to determine the IC_50_ for the antioxidant activity of TRFRB and GT3, the rat liver microsomes were pretreated with 0.5, 1, 5, 10, 50, 100, and 500 *μ*M of pure GT3 or TRFRB for 1 h. The microsomes were then subjected to a powerful oxidant TBHP for an additional 30 min. IC_50_ values for both are calculated as the concentration that reduced the amount of lipid peroxidation approximately 50%. The IC_50_ values were 5.3 *μ*M and 10.3 *μ*M for pure GT3 and TRFRB, respectively, suggesting a comparable antioxidant capacity of TRFRB similar to pure GT3 against TBHB induced lipid peroxidation (Figure 2).

**Figure 3:**
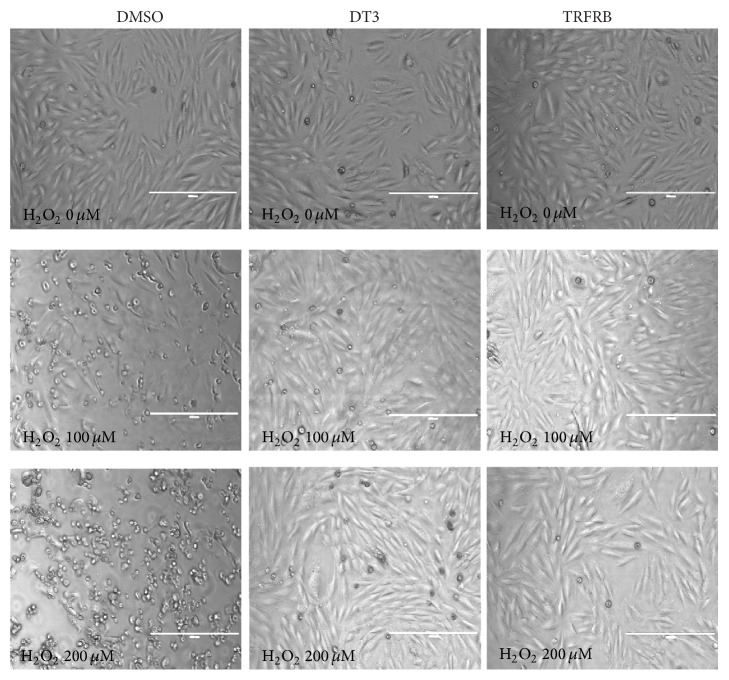
Pretreatment with 5 *μ*M DT3 or TRFRB significantly protected the cell morphology against H_2_O_2_ treatment. Pictures were taken at 10x magnification. The scale bar represents 400 *μ*m.

We then investigated whether TRFRB can protect the various cells types from different tissue origins against H_2_O_2_ or IR induced oxidative damage. Pretreatment of H9c2 rat heart cardiomyocytes with 5 *μ*M of DT3 or TRFRB significantly protected the morphology of these cells from 4 h of 100 and 200 *μ*M H_2_O_2_ treatment (Figure 3), suggesting that TRFRB is as effective as a pure tocotrienol isomer against peroxide induced cell injury.

**Figure 4:**
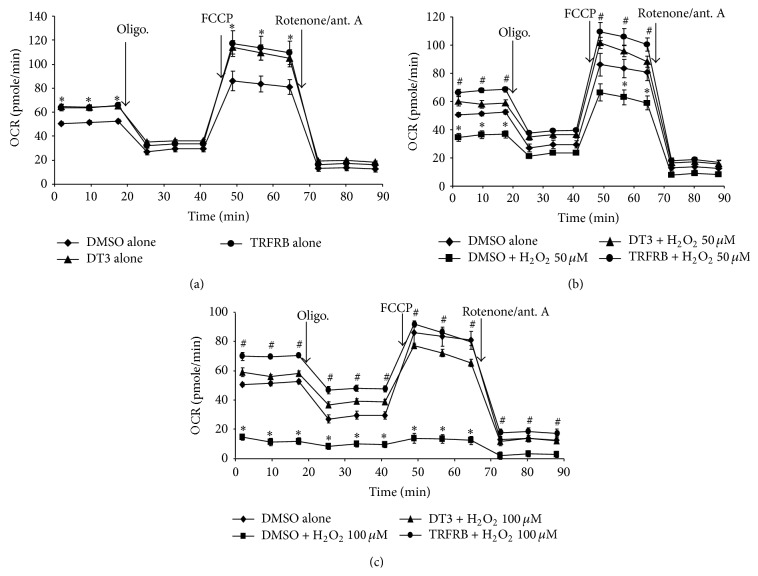
Pretreatment with 5 *μ*M DT3 or TRFRB preserved the cellular bioenergetics and mitochondrial respiration of H9c2 cells following H_2_O_2_ injury. Treatment of both DT3 and TRFRB increased basal oxygen consumption rate (OCR) as well as maximum OCR in H9c2 rat heart cardiomyocytes (a). Treatment with 5 *μ*M of DT3 or TRFRB prior to 50 (b) or 100 *μ*M H_2_O_2_ (c) resulted in complete protection and restored the mitochondrial respiration. Each data point represents mean ± SEM of *n* = 8–16 wells from 2 separate experiments. ^*∗*^ *p* < 0.01 as compared to control, ^#^ *p* < 0.001 as compared to H_2_O_2_ treated group.

We and others have previously shown that IR induced oxidative stress significantly and adversely affects mitochondrial electron transport chain and respiration [3, 7, 30]. Therefore, the next experiment assessed the effects of TRFRB and pure DT3 on cellular bioenergetics and mitochondrial respiration in the absence of any cellular stress. Intracellular mitochondrial function was examined by sequentially adding inhibitors of oxidative phosphorylation [24, 30]. Initially we took 3 measurements to determine the “basal respiration” of the cells. Next, by injecting an inhibitor of mitochondrial ATP synthase (oligomycin), a decrease in oxygen consumption rate (OCR) was obtained and this decrease in OCR is termed as “ATP linked respiration.” In order to determine the maximal respiration potential of the cells, FCCP, an uncoupler, was used. Immediately following FCCP injection, oxygen consumption increased and cells reached to their maximum respiration. Mitochondrial “reserve respiratory capacity” was calculated by subtracting the maximum OCR from the basal OCR. Overnight (16 h) treatment of both DT3 and TRFRB increased basal oxygen consumption rate (OCR) as well as maximum OCR in H9c2 rat heart cardiomyocytes (Figure 4(a)). When H9c2 cells were treated with additional 4 h of 50 *μ*M H_2_O_2_, the basal, the ATP linked, and maximum OCR were significantly decreased compared to the control group. 100 *μ*M H_2_O_2_ treatment for 4 h completely halted the cellular respiration (Figures 4(b) and 4(c)). Treatment with 5 *μ*M of DT3 or TRFRB for 16 h prior to 50 or 100 *μ*M H_2_O_2_ resulted in complete protection and restored the mitochondrial respiration of these cells (Figures 4(b) and 4(c)).

**Figure 5:**
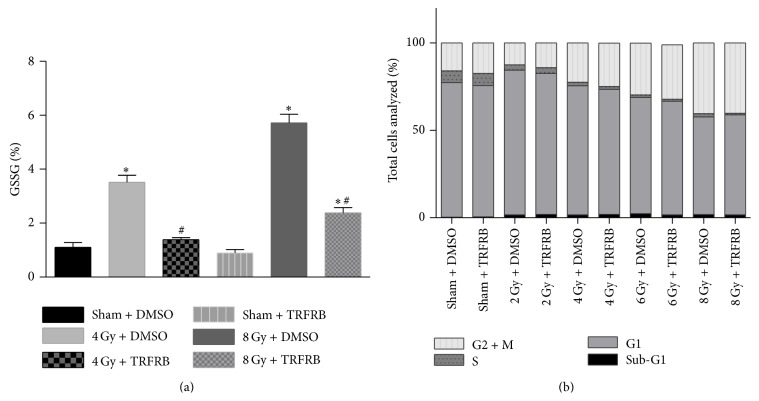
(a) Oxidized glutathione levels in dermal fibroblasts were increased at 72 h when the cells were exposed to 4 or 8 Gy IR. This increase was prevented when cells received 5 *μ*M TRFRB 24 h before IR. Each data point represents mean ± SD of *n* = 3 treatment dishes. ^*∗*^ *p* < 0.001 as compared to sham DMSO group, ^#^ *p* < 0.001 as compared to IR treated group. (b) There was no significant change in these cells that received TRFRB for the cell cycle reentry in 24 h following 2, 4, 6, or 8 Gy irradiation.

Next we extended our findings into a different cell type and different cellular injury. Since therapeutic irradiation can result in significant skin injury, we focused on two different skin cell types to determine whether TRFRB can protect human skin cells from IR induced stress. First primary human dermal fibroblast (HDF) cells were irradiated at different doses and 24 h following IR the cells were collected to measure glutathione (GSH) and glutathione disulfide (GSSG) levels. Additional 2 sets of samples were also collected to be analyzed for cell cycle or replated for clonogenic cell survival assay. Both 4 and 8 Gy IR significantly increased the percentage of GSSG over total GSH suggesting an increase in oxidative stress in HDFs at these two IR doses. Pretreatment with 5 *μ*M TRFRB reversed this effect and significantly decreased the % GSSG levels in HDFs (Figure 5(a)). TRFRB protection from IR induced oxidative stress was not extensive enough to improve HDF cells' reentry to the cell cycle (Figure 5(b)). TRFRB also did not protect HDFs from IR induced clonogenic cell killing from 2, 4, 6, or 8 Gy IR exposure (data not shown).

**Figure 6:**
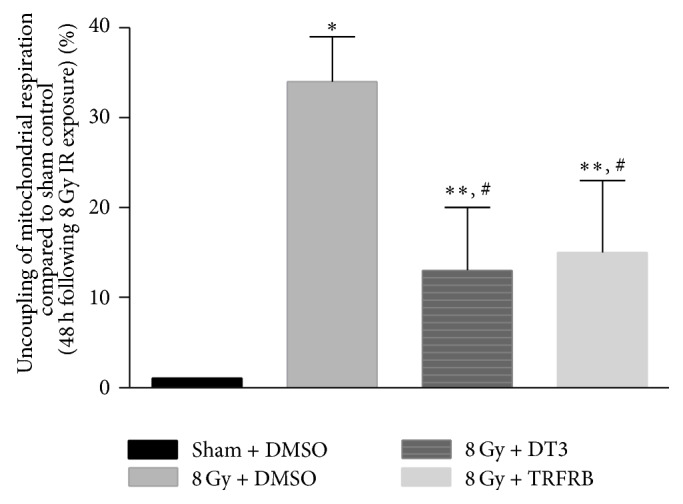
The degree of mitochondrial uncoupling of HaCaT cells was significantly increased at 48 h when exposed to 8 Gy IR and this increase was reversed in the presence of 5 *μ*M DT3 or TRFRB. Each data point represents mean ± SEM of *n* = 8–16 wells from 2 separate experiments. ^*∗*^ *p* < 0.001 as compared to sham DMSO group, ^*∗∗*^ *p* < 0.05 as compared to sham DMSO group, and ^#^ *p* < 0.05 as compared to IR treated group.

Secondly we utilized an immortalized human skin keratinocyte line (HaCaT cells) to evaluate the radioprotective effects of TRFRB. HaCaT cells were pretreated with DMSO vehicle or 5 *μ*M of DT3 or TRFRB for 16 h prior to 8 Gy IR. Then the cellular bioenergetics and mitochondrial respiration in these cells were assayed at 48 h following IR exposure as described with H9c2 cells. The percent mitochondrial uncoupling is defined and calculated by determining the increase in the ratio of basal respiration to maximum respiration (OCR following FCCP treatment) and the data was normalized to the sham irradiated control group. The degree of mitochondrial uncoupling of HaCaT cells was significantly increased at 48 h when exposed to 8 Gy IR and this increase was partially but significantly reversed in the presence of 5 *μ*M of DT3 or 5 *μ*M of TRFRB pretreatment (Figure 6).

**Figure 7:**
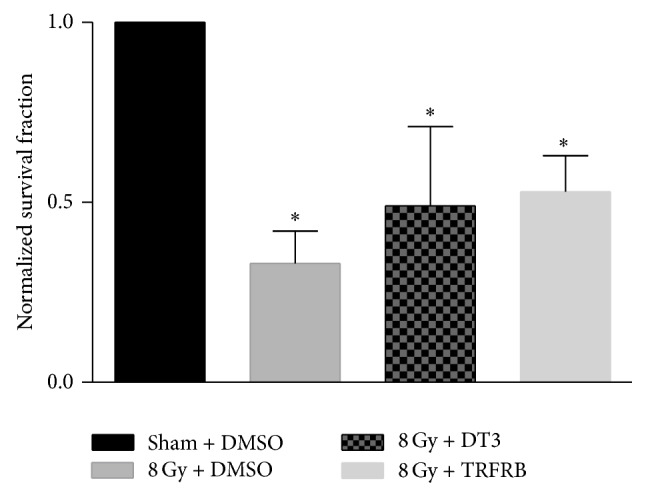
When HaCaT cells were irradiated with 8 Gy IR, received DT3 or TRFRB 4 h after irradiation, and plated for clonogenic cell survival 72 h following IR, there was an increase in cell survival in TRFRB treated cells. However, this increased survival fraction was not statistically significant compared to 8 Gy IR alone group. Each data point represents mean ± SD of *n* = 3 treatment dishes. ^*∗*^ *p* < 0.001 as compared to sham DMSO group.

**Figure 8:**
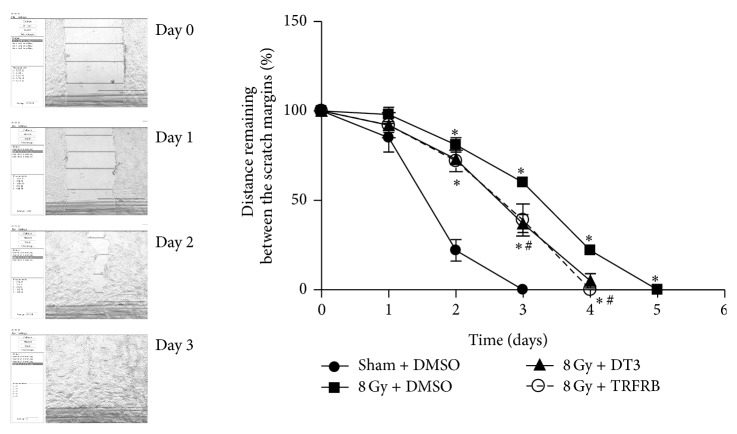
Migration rates of HaCaT cells were decreased when the cells were exposed to 8 Gy IR. This decrease was partially restored when cells received 5 *μ*M DT3 or TRFRB 4 h after IR exposure. Each data point represents mean ± SD of five points per field, with two fixed fields (*n* = 10). ^*∗*^ *p* < 0.01 as compared to sham DMSO group, ^#^ *p* < 0.01 as compared to IR treated group.

In our next set of experiments we wanted to determine whether TRFRB could protect the skin cells from IR induced injury even if TRFRB is given after IR exposure. When HaCaT cells were irradiated with 8 Gy IR, received DMSO, 5 *μ*M of DT3, or 5 *μ*M of TRFRB 4 h after irradiation, and plated for clonogenic cell survival 72 h following IR, there was a slight increase in cell survival in both DT3 and TRFRB treated cells. This protection in the increased survival fraction provided by DT3 or TRFRB was not statistically significant compared to 8 Gy IR alone group (Figure 7). An* in vitro* scratch assay was utilized to evaluate the protective efficacy of TRFRB for keratinocyte migration function when the cells were treated with DMSO, 5 *μ*M of DT3, or 5 *μ*M of TRFRB after IR exposure. Migration rates of HaCaT cells were significantly improved (although they did not completely reach the levels of DMSO vehicle group) even if the cells were treated with DT3 or TRFRB 4 h after 8 Gy IR exposure (Figure 8).

## 4. Discussion

There is a significant and unmet need for the development of alternative strategies and novel agents to alleviate radiation side effects. The only approved radioprotective agent amifostine has limited use due to its side effects [31]. Tocotrienols, vitamin E analogs, have been gaining a great deal of attention in the past decade as radioprotectors for radiotherapy patients as well as in the context of accidental exposure of large populations. Their unsaturated isoprenoid side chain differentiates tocotrienols from tocopherols, which are less efficacious in protection against ionizing radiation. In addition to their antioxidant properties, the tocotrienols have been shown to increase tetrahydrobiopterin levels and thus increase eNOS activity. They also effectively inhibit 3-hydroxy-3-methylglutaryl-coenzyme A (HMG-CoA) reductase enzyme, enhance hematopoietic recovery, reduce intestinal radiation injury, and accelerate the recovery of soluble markers of endothelial function [13, 17–20, 32, 33]. Despite their superb radioprotective effects, however, tocotrienols (DT3 and GT3) are in short supply and very expensive to purify. It is in this context that a by-product of rice oil refinement could provide a viable alternative source for isolation of tocotrienols in large quantities.

Here we provide evidence that a tocotrienol-rich fraction of rice bran oil deodorizer distillate, TRFRB, shows efficacy against peroxide and ionizing radiation induced injury* in vitro.* TRFRB demonstrated a significant antioxidant function against hydroperoxide induced lipid peroxidation in rat liver microsomes, which was comparable to that of pure GT3.

Various cultured cell models were also utilized to explore which attributes of TRFRB were required for protection against H_2_O_2_ and IR induced cell damage in cardiomyocytes, epidermal keratinocytes, and dermal fibroblasts. TRFRB demonstrated a profound effect preserving the morphology of cardiomyocytes against H_2_O_2_ exposure. TRFRB also protected the human dermal fibroblasts cells from IR induced oxidative stress by preventing the oxidation of glutathione following IR exposure. However, the reversal of oxidation of this important thiol was not enough to protect the dermal fibroblasts from IR induced cytotoxicity determined by the clonogenic cell survival assay. Interestingly, epidermal keratinocytes treated with DT3 or TRFRB after irradiation demonstrated only a slight increase in clonogenic cell survival. These results suggest that tocotrienols may protect the overall redox homeostasis in the cells but they may not be sufficient to protect from other types of IR induced injuries* in vitro* (e.g., DNA damage, modulation of cell cycle proteins such as cyclin B1).

More importantly migration rates of keratinocytes were significantly improved by DT3 or TRFRB treatment even though they received TRFRB 4 h after exposure to IR. Additional DT3 or TRFRB treatment of these cells at 36 h following radiation exposure probably fortified and protected the keratinocytes from IR induced late ROS effects. This is significant since keratinocyte migration is a crucial component of wound healing, which is known to be perturbed when skin is exposed to IR [34, 35].

Our mitochondrial function and cellular respiration studies also revealed significant effects of TRFRB on cells with or without any cellular stress. Oxygen consumption rates of the cells were measured using the XF96 Extracellular Flux Analyzer. The increases measured in basal and maximal OCR suggest that both DT3 and TRFRB enhanced cellular respiration, which could be due to an increase in mitochondrial biogenesis or enhanced function of existing mitochondria. Our calculations also demonstrated an increase in reserve respiratory capacity of the cells implying that TRFRB and DT3 improve the ability to respond against any stress that might increase the energy demand in the cell. This supposition was validated when the cells were exposed to two powerful stressors, H_2_O_2_ or IR. In both cases, pretreatment of cardiomyocytes or skin keratinocytes with TRFRB exhibited significant protection of mitochondrial function.

Collectively these results strongly suggest that the antioxidant capacity of TRFRB was critical to its ability to protect cells from oxidative injuries (via H_2_O_2_ or IR). However, our cellular bioenergetics studies also allow for the speculation that TRFRB protects the cells against IR injury by partly preserving the cells' mitochondrial function. Our future studies will include investigation of the molecular mechanisms by which TRFRB exerts its radioprotective/mitigative effects in different cell types, as well as its efficacy against IR induced normal tissue injury* in vivo*.

## Figures and Tables

**Table 1:** Chemical composition of TRFRB determined via GC/MS.

Compound	Tocopherol	Tocotrienol	Others
*δ*-Tocopherol	4.3		
*β*-*γ*-Tocopherol	11.7		
*α*-Tocopherol	9.3		
*β*-*γ*-Tocotrienol		9.2	
*α*-Tocotrienol		8.1	
Squalene			31.2
Campesterol			3.2
Sitosterol			5.2
Cycloartenol			3.7
2,4-Methylenecycloartenol			4

Total %	25.3	17.3	47.3
